# Chimeric Antigen Receptor T-cell Therapy for Chronic Lymphocytic Leukemia: What is the supporting evidence so far?

**DOI:** 10.46989/001c.88382

**Published:** 2023-11-28

**Authors:** Razan Mohty, Shaykha Alotaibi, Martha Gadd, Yan Luo, Ricardo Parrondo, Hong Qin, Mohamed A. Kharfan-Dabaja

**Affiliations:** 1 Department of Blood and Marrow Transplantation and Cellular Immune Therapy, Moffitt Cancer Center, Tampa, Fl, USA; 2 Division of Hematology-Oncology, Blood and Marrow Transplantation and Cellular Therapy Program, Mayo Clinic, Jacksonville, FL, USA; 3 Regenerative Immunotherapy and CAR-T Translational Research Program, Mayo Clinic, Jacksonville, FL, USA

## Abstract

While acknowledging that newer therapies have improved survival rates in chronic lymphocytic leukemia (CLL), patients with high-risk disease features are at an increased risk of treatment failure. Allogeneic hematopoietic cell transplantation (allo-HCT) was traditionally offered as front-line consolidation in high-risk CLL; however, with the emergence of targeted therapies like Bruton tyrosine kinase (BTK) and B-cell lymphoma 2 (BCL-2) inhibitors, the role of allo-HCT has been relegated to later stages of the disease. Patients with relapsed/refractory (R/R) CLL who have failed both BTK and BCL-2 inhibitors represent a therapeutic challenge owing to a poor prognosis. Chimeric antigen receptor T-cell (CAR T) therapies targeting CD19 have improved response rates and overall survival in various types of R/R B-cell non-Hodgkin lymphomas. For CLL, no approved CAR T-cell therapies are yet available. Emerging data appear to show a therapeutic benefit of CAR T-cell therapy in patients with R/R CLL, even after failing an allo-HCT.

## INTRODUCTION

It is estimated that 18,740 chronic lymphocytic leukemia (CLL) cases will be diagnosed in the United States of America in 2023, representing one of the most common leukemias in incidence and prevalence.^1^ Unfortunately, it is also anticipated that 4,490 cases will die from CLL this year despite remarkable therapeutic advances.^1^ In recent years, treatment of CLL has moved away from traditional chemoimmunotherapy, which combined bendamustine plus rituximab (BR) or a more intense combination of fludarabine, cyclophosphamide and rituximab (FCR), or other variations incorporating a newer generation of CD20 monoclonal antibodies such as obinutuzumab or ofatumumab, to therapies targeting the Bruton tyrosine kinase (BTK) or B-cell Lymphoma 2 (BCL-2).^2,3^ These have revolutionized the treatment of CLL when prescribed as monotherapy, as is the case of BTK inhibitors (BTKi), or by combining a BTKi, such as ibrutinib, with the BCL-2 inhibitor, venetoclax.^4,5^ Also, striking results have been reported when combining venetoclax with anti-CD20 monoclonal antibodies.^6,7^ Nowadays, the anticipated 5-year survival exceeds 70% for patients with CLL.^8,9^

Despite these advances, patients harboring adverse chromosomal aberrations such as del17p with or without complex karyotype, mutant *TP53*, those with unmutated immunoglobulin heavy chain gene *IGHV,* or other abnormalities^10^ are at a high(er) risk of relapse even when treated with BTKi.^11^ Accordingly, these cases represent a real therapeutic challenge. Allogeneic hematopoietic cell transplantation (allo-HCT) used to be offered as front-line consolidation to high-risk CLL patients when chemoimmunotherapy was the main front-line treatment.^12,13^ With the emergence of BTKi and BCL-2 inhibitors, the role of allo-HCT became more limited and relegated to later stages of the disease.^14^ However, there appears to be a renewed interest in allo-HCT for patients with CLL who do not respond or who relapse after BTKi and/or BCL-2 inhibitors.^15^ While reduced intensity conditioning (RIC) regimens improved tolerability of allo-HCT in this population of patients with generally advanced age and existing comorbidities, applicability of the procedure remains limited, due to toxicities, mainly acute and chronic graft-versus-host disease (GVHD), organ failure, infections, and resulting non-relapse mortality.^16,17^

Chimeric antigen receptor T-cell (CAR T) therapy epitomizes the successful advances in cancer immunology and T-cell engineering. These therapies have modernized the treatment algorithms of various types of B-cell non-Hodgkin lymphomas (NHL) and B-cell acute lymphoblastic leukemia (ALL).^18–22^ Axicabtagene ciloleucel (axi-cel), tisagenlecleucel and lisocabtagene maraleucel (liso-cel) are approved for treatment of patients with relapsed and/or refractory (R/R) diffuse large B-cell lymphoma (DLBCL) who failed 2 or more lines of systemic therapy, including those who had transformed follicular lymphoma (tFL).^18–20^ Axi-cel and liso-cel are also approved when DLBCL fails to respond to front-line anthracycline-based chemoimmunotherapy, or if the relapse occurs within 12 months from its completion.^23,24^ Axi-cel and tisagenlecleucel are approved for patients with R/R FL after 2 lines of therapy^25,26^; and brexucabtagene autoleucel (brexu-cel) is approved for R/R mantle cell lymphoma (MCL) and B-cell ALL based on results of the ZUMA-2 and ZUMA-3 studies, respectively.^22,27^ Tisagenlecleucel is a CAR T-cell therapy that is approved for R/R B-cell ALL, patients up to 25 years of age.^21^

Although single case reports and small single-institution studies have demonstrated the feasibility of CAR T-cell therapy for the treatment of CLL, no approved CAR T-cell therapies are available yet.^28–30^ Promising data are emerging on the results of a multicenter phase 1 clinical trial for the treatment of high-risk R/R CLL patients.^31^ In this review, we provide a comprehensive appraisal of the literature on CAR T-cell therapy in treating CLL.

### CD19 directed CAR T-cell therapy for CLL

The prognosis of patients with R/R CLL who have been treated with both a covalent BTKi and a BCL-2 inhibitor (venetoclax, in particular) is relatively poor with currently available therapies. Two retrospective studies were conducted to investigate the outcomes of these patients. The first included 33 patients, 11 with double-refractory disease (refractory to ibrutinib and venetoclax). The median treatment-free and overall survival (OS) to subsequent therapy that combined ibrutinib and venetoclax were only 11.2 and 27.0 months, respectively.^32^ In the second retrospective study of 125 patients with CLL previously exposed to a covalent BTKi and venetoclax, the overall response rates (ORR) and progression-free survival (PFS) estimates to subsequent therapies ranged from 31% to 76% and 3 to 14 months, respectively; however, the median PFS for patients who discontinued a previous covalent BTKi for progressive disease was only 1 month, versus 7 months for those who had to discontinue due to adverse events.^33^ These data highlight the urgent need for novel therapies for double-refractory CLL patients.

CAR T-cells are genetically engineered T-cells which combine a single-chain variable fragment (scFv) domain of a targeting antibody with intracellular signaling and costimulatory domains. Anti-CD19 CAR T-cell therapy has revolutionized the treatment of R/R B-cell lymphoid malignancies, improving objective response rates and survival outcomes.^18–21^ Emerging data are also beginning to demonstrate the therapeutic benefit of anti-CD19 CAR T-cell therapy for R/R CLL.

In a pilot/phase I study, CTL019, an anti-CD19 CAR T-cell, was evaluated to determine its safety, efficacy, and cellular kinetics in chemotherapy-resistant or refractory CD19+ CLL.^34^ Of 23 patients enrolled, 14 received CTL019. The median age was 66 years. Patients had received a median of 5 previous lines of therapy, and 6 patients had del17p or *Tp53* mutation. Only one patient had progressed on prior ibrutinib, and none were exposed to venetoclax. Lymphodepleting chemotherapy regimens included fludarabine/cyclophosphamide (Flu/Cy) (n=3), pentostatin/cyclophosphamide (n=5), and bendamustine (n=6). Responses were determined based on the 2008 International Workshop on CLL (iwCLL) criteria.^35^ After a median follow-up of 19 months, 8 (57%) of 14 patients had an objective response, with 4 (29%) achieving a complete response (CR). No patient with CR appeared to have relapsed, with median duration of response (DOR) of 40 months. Four (29%) patients achieved a partial response (PR) within the first month of CTL019 infusion, with a median DOR of 7 months. The estimated median OS was 29 months, and the 18-month OS was 71%. The estimated median PFS was 7 months, with an 18-month PFS of 28.6%.^34^ CAR T-cell kinetics evaluation revealed a more robust expansion of CAR T-cells in patients who achieved CR compared to those without similar hemophagocytic lymphohistiocytosis-like syndrome. The median onset of cytokine release syndrome (CRS) was 7 days after CTL019 infusion. Only one patient developed a grade 3 neurologic event. Finally, one patient in CR died 21 months after infusion due to infection-related complications. This study shows that CAR T-cell therapy for patients with R/R CLL is feasible, albeit at an increased risk of severe CRS. This highlights the need for future studies to determine predictors of high toxicity and assess the optimal preventative intervention.

In a prospective phase I/II trial (NCT01865617), another anti-CD19 CAR T-cell construct was evaluated in R/R CLL. A total of 24 patients (Richter transformation=5) were included. All patients received Flu/Cy lymphodepletion.^29^ CAR T-cells were administered at 3 dose levels (DL): DL1 (2 x 10^5^ CAR T-cells/kg), DL2 (2 x 3 10^6^ CAR T-cells/kg), or DL3 (2 x 3 10^7^ CAR T-cells/kg. Four patients received DL1, 19 DL2, and one DL3. At 4 weeks after infusion, the ORR by the 2008 iwCLL criteria^35^ was 71% (CR, 4/19, 21%; PR, 10/19, 53%). The median PFS was 8.5 months, and the median OS was not reached. Of 24 patients, the majority (83.3%) experienced CRS, yet only 2 (4.1%) had ≥grade 3 CRS, one being fatal. Neurotoxicity was observed in 8 (33%), with 6 (25%) having ≥grade 3 neurotoxicity including 1 fatal case. In this study, achieving an immunoglobulin heavy locus (IGH)-negative status (versus CR) after CAR T-cells demonstrated a stronger correlation with prolonged PFS. While most patients with bone marrow involvement responded to CAR T-cell therapy, complete elimination of bulky nodal disease was less commonly reported.^29^ Overall, the results of this study suggest that anti-CD19 CAR T-cell therapy for R/R CLL is effective and promising.

Recent results from the phase-1 dose-escalation portion of a multicenter, open-label, phase 1/2 TRANSCEND CLL 004 (NCT03331198) study of liso-cel reported promising efficacy.^31^ A total of 177 patients, at median age of 65 years, with R/R CLL were included, and 83% of patients had high-risk genetic features, including *TP53* mutation, del17p, complex karyotype, and/or unmutated *IGHV*. All patients had received prior BTKi. Patients received DL 1 (50x10^6^ CAR+ T-cells) or DL2 (100x10^6^ CAR+ T-cells). Efficacy was evaluable in 96 (DL1 = 9; DL2 = 87). Responses were reported as per the 2018 iwCLL guidelines^36^ after a median follow-up of 21.1 months. The ORR was 47.1%, and the CR/CR with incomplete marrow recovery (CRi) rate was 18.4%. The median DOR was 35.3 months, and the median PFS was 18 months. Overall, 56 (64.4%) patients achieved undetectable minimal residual disease (uMRD) (sensitivity,10^-4^) in peripheral blood; and 51 (58.6%) also achieved uMRD in the bone marrow. The CRS rate was 84.6% (grade 3=8.5%), and 45.3% of the patients had neurological events (NE) (grade 3=17.9%, grade 4=0.9%). Prolonged cytopenia and hypogammaglobulinemia occurred in 53.8% and 15.4%, respectively. One patient died of hemophagocytic lymphohistiocytosis.^37^

These results show the efficacy and tolerability of anti-CD19 CAR T-cell therapy for patients with R/R CLL, even in those with high-risk CLL. **Table 1** showcases several smaller studies with less than 10 patients. Nonetheless, longer follow-up periods from these early-phase trials and confirmatory phase 3 trials are necessary to validate these findings. Still, a number of these studies showed high rates of severe CRS. This emphasizes the need to better understand and identify toxicity predictors and develop strategies to mitigate them. There is potential for further improvement of anti-CD19 CAR T-cell therapy efficacy through various approaches, such as administering a second infusion or combination of CAR T-cells with other anti-CLL agents such as BTKi, BCL-2 or PI3 kinase inhibitors.^38,39^

**Table 1.** Studies Evaluating Single-Agent Anti-CD19 CAR T-cell in patients with CLL

**Reference**	**Study Type**	**N**	**All-grade CRS** **n/N (%)**	**All-grade Neurotoxicity n/N (%)**	**ORR** **(%)**	**CR** **(%)**	**Follow-up, median (range)**
Brentjens et al^40^	Phase I/II	8	7 (87.5%)	0	0	0	NR
Kalos et al^41^	Phase I	3	3/3(100%)	0	100%	66%	NR
Kochenderfer et al^42^	Phase I/II	4*	5/⁠8 (62.5%)	0	75%	25%	NR
Cruz et al^43^	Phase I	4	0	0	25%	None	NR
Kochenderfer et al^42,44^	Phase I/II	5*	3/5 (60%)	1/5 (20%)	100%	60%	NR
Brudno et al^45^	Phase I	5	4/5 (80%)	0	40%	20%	NR
Ramos et al^46^	Phase I	2	NR	NR	0	0	NR
Geyer et al^47^	Phase I	8	4/8 (50%)	0	62.5%	37.5%	57.9 months
Cappell et al^48^	Phase I	8*	NR	NR	88%	63%	42 months
Gauthier et al^38^	Phase I/⁠II	9^#^	NR	NR	33.3%	22.2%	18 months

Abbreviations: BAFF-R: B-cell activating factor receptor; CD: cluster of differentiation; CLL: chronic lymphocytic leukemia; CR: complete remission; CRS: cytokine release syndrome; EFS: event free survival; N: number of patients; NR: not reported or not reached, NE: ORR: overall response rate; OS: overall survival; PFS: progression-free survival.

*Denote patient overlap between studies

<sup>#</sup> Second infusion of anti-CD19 CAR T-cell

### Combining BTK inhibitors and CAR T-cells

Despite the observed response and efficacy of CAR T-cell therapy in patients with CLL, long-term data regarding durability of responses are scarce. Several factors affect responses after CAR T-cell therapy. In CLL, 2 should be highlighted: T-cell dysfunction prior to CAR T-cell manufacturing and the immunosuppressive tumor microenvironment in CLL. These limit *ex-vivo* expansion and, ultimately, response to CAR T-cell therapy. T-cells derived from CLL patients exhibit exhaustion phenotype, depicted by the expression of inhibitory markers, functional defect in proliferation and cytotoxicity of CD8+ T-cells, and Th2-polarized immune response.^49,50^ It has been hypothesized that ibrutinib might improve T-cell function in CLL patients.^51^ In addition to BTK inhibition, ibrutinib exhibits an off-target effect through inhibition of IL-2 inducible T-cell kinase (ITK), restoring Th1 immune response and rebalancing the immune system.^50^ *Fraietta et al* showed that T-cell function is restored following a prolonged treatment with ibrutinib (5-11 cycles) when cultured; and that CAR T-cell expansion is improved with concomitant treatment with ibrutinib in xenograft mice models.^51^

In addition to improving T-cell function and CAR T-cell expansion, concomitant ibrutinib therapy could help mitigate toxicity.^51^ BTK plays an important role in the tumor microenvironment and promotes pro-inflammatory responses.^52^ Inhibition of BTK with ibrutinib or acalabrutinib has decreased pro-inflammatory cytokine secretion.^53^ Conversely, zanubrutinib did not exhibit similar results *in vitro* or mice when combined with CAR T-cells, compared to ibrutinib.^54^ These findings may be attributed to the uniquely distinctive characteristics of ibrutinib in modulating tumor microenvironment and improving T-cell function, as well as BCR signaling pathway inhibition.^54^

*Gauthier et al.* reported outcomes of 19 patients treated with CAR T-cell therapy with concurrent ibrutinib in a phase 1/2 trial **(Table 2)**.^55^ Patients were intended to be treated with ibrutinib 420 mg ≥2 weeks prior to apheresis and to continue until 3 months after CAR T-cell therapy. All had been exposed to ibrutinib prior to CAR T-cell. Deep responses were observed with ibrutinib combination: 15 (83%) responses per iwCLL criteria and 13 (72%) MRD-negative marrow responses. This cohort was compared to a group of patients previously treated in the same trial but without ibrutinib.^55^ The 1-year OS and PFS were 64% and 38%, respectively. The addition of ibrutinib was significantly associated with lower grade CRS (median grade 1) compared to no ibrutinib (median grade 2), *p*=0.04. However, no difference in the severity of neurotoxicity between the two cohorts was described. One patient died 4 days after CAR T-cell infusion from presumed ibrutinib-related cardiotoxicity. A significantly lower lactate dehydrogenase (LDH) level was reported in the ibrutinib versus the no ibrutinib cohort (*p*=0.04). This association between the continuation of ibrutinib and lower LDH levels underscores the potential risk of disease flare-ups that may occur when the medication is abruptly discontinued.^55^

**Table 2.** Summary of a trial evaluating the combination of ibrutinib with CAR T-cell in patients with CLL

**Study**	**Study type**	**Target**	**N**	**Adverse events**	**ORR** **(%)**	**CR** **(%)**	**PFS**	**OS**
Gauthier et al^55^	Retrospective	CD19	17	CRS: 76% ≥grade 3: 0%	83%	71%	1-yr=38%	1-yr=64%
TRANSCEND CLL 004^56^	Phase 1/2	CD19	23	CRS: 74% ≥grade3: 6%	82%	45%	NR	NR
UPENN study^57^	Phase 2	CD19	19	CRS: 95% ≥grade3*: 11%	N/A	44%	2-⁠yr=80%	2-⁠yr=84%

Abbreviations: CD: cluster of differentiation; CLL: chronic lymphocytic leukemia, CR: complete remission; CRS: cytokine release syndrome; N: number of patients; NA: not applicable; NR: not reported; ORR: overall response rate; OS: overall survival; PFS: progression-free survival; yr: year; * ASTCT: (American Society for Transplantation and Cellular Therapy) grading

In another phase 2 prospective trial, *Gill et al.* investigated the safety and efficacy of combining anti-CD19 CAR T-cell therapy with a humanized binding domain (huCAR T) and ibrutinib in R/R CLL after at least 6 months of ibrutinib **(Table 2)**.^57^ A total of 20 patients were screened, and 19 were eventually infused. Patients continued ibrutinib until intolerable toxicity, or at the patient’s and/or investigator’s discretion, if MRD-negative CR for at least 6 months was attained. The median time from ibrutinib therapy to huCAR T infusion was 14 (7-50) months. At the last reported follow-up, only 5 patients had remained on ibrutinib. The causes of discontinuation were drug-related toxicity, MRD-negative CR, progression of CLL, or evidence of a secondary malignancy. At 12 months, the CR rate was 50%.^57^ Overall, 13 (72.2%) of 18 patients had uMRD. At 48 months, the estimated probability of PFS and OS were 80% and 84%, respectively. Eighteen of 19 patients developed CRS, with 63.5% of them being grade 1. Five patients developed neurotoxicity, with 1 of them developing grade 4 Immune tell-associated neurologic syndrome (ICANS). One patient died of grade 4 CRS and grade 4 ICANS after 10 days from CAR T-cell infusion. Another died while in CR, almost 3 years after CAR T-cell infusion, due to severe infection in the setting of hypogammaglobulinemia.^57^

Early reports from the phase-1 cohort of the TRANSCEND CLL 004 trial of CAR T-cell therapy for CLL in combination with ibrutinib also showed promising results **(Table 2)**.^56^ The patients continued ibrutinib during leukapheresis and at least 90 days after infusion. A total of 19 CLL (high-risk cytogenetics=95%) patients were included. All patients were R/R to ibrutinib. Toxicities attributable to ibrutinib were observed in 13 patients (diarrhea=7, hypertension=4, atrial fibrillation=1, and skin rash=1). Fourteen developed CRS (grade 3=1). Neurologic events occurred in 6 patients, with 3 of them being ≥grade 3. The 3-month ORR was 83%. Peripheral blood MRD-negative remission was reported in 89%.^56^

These studies demonstrate the safety and efficacy of adding BTKi to CAR T-cell therapy in R/R CLL. This combination appears to improve efficacy and mitigates toxicity of CAR T-cell. Larger studies with longer follow-up are needed to confirm these findings and to determine the optimal duration of BTKi combination therapy.

### Other targets beyond CD19

As aforementioned, anti-CD19 CAR T-cell therapy in patients with R/R CLL has not yet shown the efficacy observed in other B-cell lymphoid malignancies. A recent evaluation of a CD19 CAR T-cell therapy clinical trial highlighted only 26% of CLL patients showing a durable response versus the 90% complete remission observed in ALL.^58^ This has led to the proposal of several approaches, including novel targets, progressive CAR designs, and innovative treatment regimens. We present some novel targets being explored in CLL related to CAR T-cell therapy designs **(Table 3)**.

**Table 3.** Ongoing trial evaluating novel/dual/multi-targets for CAR T-cell therapy in CLL

**Study Design**	**CAR T construct**	**Target population**	**Status**	**Clinicaltrial.gov identifier**
Phase I/II	CD20-sepcitic CAR T-cells	R/R B-cell NHL	Recruiting	NCT03277729
Phase I	Anti-CD19/CD20/CD22 CAR T-cells	R/R lymphoid malignancies	Recruiting	NCT05418088
Phase I/II	Dual anti-CD19/CD22 CAR T-cells	CD19/CD22 positive leukemia or lymphoma	Recruiting	NCT04029038
Phase I	Dual anti-CD19/CD22 CAR T-cells	CD19/CD22 positive B-cell malignancies	Recruiting	NCT03448393
Phase I	Anti-ROR1 CAR T-cells	Advanced ROR1 positive malignancies	Terminated due to slow accrual	NCT02706392
Phase I	Anti-CD37 CAR T-cells	Hematologic malignancies	Recruiting	NCT04136275
Phase I	Anti-Kappa-CD28 CAR T-cells	NHL, CLL or MM	Recruiting	NCT00881920
Phase I	Anti-Kappa-CD28 CAR T-cells	NHL, CLL/SLL	Recruiting	NCT04223765
Phase I	Anti-BAFFR-CAR T-cells	R/R B-cell NHL	Recruiting	NCT05370430

Abbreviations: CAR: chimeric antigen receptor; CD: cluster of differentiation; CLL: chronic lymphocytic leukemia; MM: multiple myeloma; NHL: non-Hodgkin lymphoma; ROR1: receptor tyrosine kinase-like orphan receptor 1; R/R: refractory or relapsed; ; SLL: small lymphocytic lymphoma;

CD20 is a cell surface protein expressed on B-cells along the many stages of B-cell development, similar to CD19. Targeting CD20 with anti-CD20 monoclonal antibodies started with rituximab in 1997.^59^ The surface expression of CD20 can be downregulated after prolonged exposure to CD20 targeted therapy through clonal evolution or epigenetic mechanisms^60^; however, rituximab is still a ready addition to current treatment regimens, such as with venetoclax in R/R CLL.^61^ Furthermore, CD20 CAR T-cell engineering has generated encouraging results in clinical trials^62,63^; in fact, CD20 CAR T-cell therapy was applied to R/R B-NHL with favorable outcomes,^64^ and circulating or residual rituximab may not negatively impact CD20 CAR T-cell efficacy.^65^

CD22 is a sialoglycoprotein that is expressed on immature B-cells through to mature B-cell development and has four immunoreceptor tyrosine-based inhibitory motifs (ITIMs) in its cytoplasmic tail, which serve to inhibit B-cell receptor signaling. Genetic CD22 variants in humans have been linked to susceptibility to autoimmune diseases.^66^ As for CD20, a family of anti-CD22 monoclonal antibodies has been generated with the goal to treat B-cell pathologies, specifically autoimmune diseases^66^ and malignancies. CD22-ligand binding induces internalization, which makes it a strong target for antibody-drug conjugates and radioimmunoconjugates.^67^

B-cell activating factor receptor (BAFF-R) (B lymphocyte stimulator, BLyS, CD257, TALL-1, or TNFRSF13C) plays a seminal role in B-cell maturation and differentiation, such that without BAFF-R there are no mature B-cells. BAFF-R works with TACI, another survival receptor, to regulate antibody production, prevent apoptosis, and enhance mitochondrial function to increase life span.^68^ As the BAFF ligand binds to BAFF-R, the non-canonical NF-kB2-dependent pathway is engaged to initiate survival cascades,^69^ and overlaps with many BCR signaling pathways.^70^ In the case of malignancy, this powerful survival pathway allows for the survival and accumulation of tumor cells. Anti-BAFF-R CAR T-cell therapies have already been tested *in vitro* and *in vivo* models and showed robust efficacy in xenograft mice models.^71,72^ Clinical trials are actively recruiting for the treatment of R/R B-cell ALL (NCT04690595) and R/R B-cell NHL (NCT05370430) thus, application to CLL is expected.

Receptor tyrosine kinase–like orphan receptor 1 (ROR1) was identified, with its sibling ROR2, 20 years ago, during exploration of proteins with tyrosine kinase domains. However, their ligand was unknown until 2008, when wingless-related integration site (Wnt) factor Wnt5a was identified as a ligand for ROR1.^73^ A series of knock out mouse studies showed that RORs have a significant role in embryonic development.^74^ The WNT/ROR signaling cascade was initially viewed as a marker of cancer and not a target. When exploring the gene profiles of CLL cells from different patients for commonalities, *ROR1* was identified^75^; the expression of ROR1 was confirmed on the surface of CLL cells as well as in the sera of some patients.^73,76^ Originally viewed as a marker, with high levels of ROR1 associated with aggressive CLL, the accumulating data related to the ROR1 in cell signaling cascades have propelled it as a new target for developing immunological therapies, like monoclonal antibodies, such as zilovertamab, and CAR T-cell therapy. Zilovertamab (formerly known as cirmtuzumab) is showing a favorable safety profile and is also combined with other inhibitors that inhibit Bruton tyrosine kinase or BCL2.^77,78^ This is interesting, since very low levels of ROR1 have been reported in a variety of tissues,^74^ but no expression is found in mature B-cells, T-cells, monocytes, and natural killer cells.^79^ Furthermore, ROR1 is extensively and diversely glycosylated, depending on the cancer patient.^79^ With monoclonal antibody development, CAR T-cell engineering as either standard CAR technology^80,81^ or a switchable CAR-T system is not far behind to specifically target B-cell malignancies.^82^ Also, data on CAR T-cell safety testing in primates that share ROR1 homology have been encouraging; however, on-target toxicity is a concern that needs to be explored with each new ROR1 antibody or CAR construct.^82^

CD37 is a member of the tetraspanin superfamily, and is involved with several lymphocyte functions, including survival, proliferation, adhesion, and migration. CD37 is expressed on mature B-cells, with a minor expression on plasma cells, and also in several B-cell malignancies, including CLL.^83,84^ CD37 has also been noted in peripheral T-cell lymphoma samples, positioning it as a target for both B- and T-cell lymphomas.^85^ CD37 can facilitate the docking of monoclonal antibodies; however, CD37 now has a more active role in signaling by acting as a hub for the clustering of signaling complexes.^86^ The significance of CD37 became more noticed when it was examined in the context of B-cell tumorigenesis. Additionally, CD37 is important for the interaction between T- and B-cells in the release of IgG,^87^ which may be related to the tetraspanin family’s involvement in extracellular vesicle formation.^86^ Several monoclonal antibodies have been generated and tested against B-cell malignancies^86^; an afucosylated version of SMIP-16^88^ (the progenitor of TRU-016 that was renamed to otlertuzumab) was shown to have enhanced ADCC function against CLL cells, compared to the wild type SMIP-16 and rituximab.^89^ Anti-CD37 CAR T-cells are under development for both B- and T-cell lymphomas.^83^ They showed efficacy against CLL cell lines.^83,84^

CD38 has been known since 1980. It is a bifunctional enzyme that exhibits ADP-ribosyl cyclase and cADP-ribose hydrolase activities. Since then, more information has emerged on the importance of CD38, specifically on its loss resulting in impaired immune responses, metabolic disturbances, and behavioral modifications in mice, and on it being also a marker of human leukemias and myelomas.^90^ The expression pattern of CD38 in lymphocytes depends upon the cell types, the stage of development of those cells, and the degree of activation.^90^ The observation of elevated CD38 on CLL cells was, in part, due to pathways involving INFγ, JAK1/JAK2, and T-bet.^91^ Of equal note, CD38 has NADase activity, which has a profound effect of T-cell functions, due to the numerous signaling and metabolic pathways that utilizes NAD, making elevated CD38 a disruptor of NAD+ metabolism.^92^ NAD+ pools can be degraded to adenosine, which results in impaired mitochondrial and effector T-cell function; one may hypothesize that these effects contribute to the immunosuppressive characteristic of CLL, specifically escape from PD-1/PD-L1 blockade.^93^ Treatment with monoclonal anti-CD38 results in a decrease of CD38+ cells such as Bregs, Tregs, and specific T-cell populations, but this therapy can restore CD8+ cell responses.^94^ So far, the majority of CD38 CAR T-cell treatments are designed to treat multiple myeloma (MM); however, favorable safety data will allow for the segue into other hematological malignancies that are CD38+.

The targets discussed thus far have been generally B-cell markers shared across many stages of B-cell development. The mutational status of the *IGHV* correlates with the clinical outcome of patients with CLL, with the unmutated *IGHV* leading to a poorer prognosis than the mutated *IGHV*. With these observations, exploratory attention began to focus on CLL immunoglobins, due to the clonal characteristic of CLL B-cells,^95^ and this led to the identification of several targets that are either part of the immunoglobulin family or are receptors for immunoglobulins. Most directly, CAR T-cells have been generated against kappa or lambda light chains, showing favorable effects in the in vitro, in vivo, and phase I clinical trials.^96–98^

CD23 on the surface of B-cells can bind to soluble IgE or IgE-antigen complexes; this interaction has been explored in allergic and autoimmune diseases. CD23 is an Fc receptor specific for IgE (FcεRII) that is not only expressed on the surface of B-cells but also on T-cells, follicular dendritic cells, macrophages, NK cells, eosinophils, and platelets, and that negatively regulates BCR signaling.^99^ Its overexpression was associated with CLL, but initially viewed as only a prognostic marker.^100,101^ However, with the advances in CAR technology, CD23 CAR T-cells are now being explored for treating CLL.^102^ CD23 CAR T-cells appear to specifically target tumor cells which have elevated CD23 expression while sparing the majority of normal B-cells which have low CD23 expression. Furthermore, the CD23 CAR T-cell expansion was noted in the in vivo models .^103,104^

Microarray analysis of malignant B-cells by unit expression transformation assays identified TOSO as a novel protein overexpressed in CLL.^105^ TOSO is also known as Fas-inhibitory molecule 3 and as the FcR for IgM (FcμR) which is expressed on B-, T- and NK cells. FcμR participates in the internalization of IgM, which then intersects TLR and BCR pathways that allow for malignant B-cell survival.^106^ FcμR CAR T-cell therapy is an attractive option, since it targets a CLL-selective marker while sparing healthy B-cells, and serum levels of FcμR do not block FcμR CAR T-cell efficacy.^104^

## DISCUSSION AND FUTURE DIRECTIONS

In summary, novel targeted therapies, including BTKi and BCL-2 inhibitors, have supplanted conventional chemotherapy in patients with CLL. Still, patients with adverse chromosomal aberrations and high-risk mutations are at high(er) risk of relapse, even with the improved survival outcomes afforded by novel targeted therapies. Allo-HCT is nowadays relegated to later lines of therapy, usually for patients who are refractory to BTKi and BCL-2 inhibitors^14,15^ While there is the emerging option of the non-covalent BTKi pirtobrutinib after progression on covalent BTKi, pirtobrutinib affords a median PFS of only 19.4 months in CLL/small lymphocytic leukemia (SLL) patients.^107^

More recently, CAR T-cell therapy has demonstrated safety and feasibility in treating CLL, but it is not yet approved in this disease. In the recently reported phase 1/2 TRANSCEND CLL 004 trial, CAR T-cell therapy showed efficacy and manageable toxicity (the best ORR was 80% and CR/CRi rate was 60%).^37^ Yet, it is important to note that cytopenia, including neutropenia, thrombocytopenia, and anemia, has been reported as a common adverse event associated with CAR T-cell therapy.^108^ In the TRASCEND CLL 004 trial, prolonged cytopenia were reported in 53% of the CLL patients, with 17.1% grade ≥3 infections.^37^

CAR T-cell therapy has an advantage over other targeted agents because it is time-limited. Yet, one of the major causes of a relatively lower efficacy vis-a-vis the efficacy observed in other B-cell lymphoid malignancies is believed to be T-cell dysfunction in CLL.^51,109^ As aforementioned in this review, ibrutinib has been shown to help restore T-cell function by reducing T-cell exhaustion, increasing T-cell activation, and improving the microenvironment in CLL.^51^ It can reduce the levels of pro-inflammatory cytokines and chemokines, as well as increase the levels of anti-inflammatory cytokines, which makes it a good partner for a combinatorial approach to improve CAR T-cell function and to decrease toxicity. Yet, the best timing to start ibrutinib and the duration of therapy remain undefined. The role of other BTKi in combination with CAR T-cell therapy has been explored in the pre-clinical, showing limited benefit.^54^ This could be explained by the non-BTKi related properties concerning the effect on ITK and tumor microenvironment. Other combinatorial approaches that have been studied in high-grade lymphomas involve PD-1 inhibitors. However, the results have been relatively substandard, as studies evaluating the use of checkpoint inhibitor therapy after CAR T-cell failure for aggressive B-cell lymphomas yielded ORR of 19%, CR rate of 10%, and short DOR (median of 221 days).^110^

In the reported literature on anti-CD19 CAR T-cell therapy for CLL, responses appear to be promising, yet could be improved. In addition to defective T-cell function, other purported mechanisms of resistance to anti-CD19 CAR T-cell therapies include loss of target antigen, expression of inhibitory ligands (PD-L1), lack of costimulatory ligand (CD58) and resistance to immune killing.^111^ A possible way to overcome these resistance mechanisms may be to target other antigens on the CLL cell surface, including CD20, BAFF-R, CD23, and others. Multitarget CAR T-cell therapy (dual and triple) has also been explored in CLL to improve efficacy and overcome some of the resistance mechanisms, such as loss of target antigen. Many multi-antigen CAR designs have CD19 combined with CD20^112,113^ or CD22^114,115^ and even a CD19/CD20/CD22 CAR combination.^116^ CD19/BAFF-R CAR T-cells have been generated and are being evaluated in B-ALL models.^117^ As the new targets are tested in phase I clinical trials, one anticipates to see them combined with either CD19 or other targets with the goal to address antigen escape relapses. Another strategy, particularly for CLL, is to include agents that will improve function of CAR T-cells in the immunosuppressive environment that characterizes this leukemia. Engineering CARs to also express cytokines such as IL-7, IL-12, IL-15, or IL-18 is under active investigation, because of the well-known effects of cytokines on immune cell function which are critical for CAR T-cells to function properly once infused.^118–122^

Other treatment options for CLL in the horizon are bispecific T cell engagers (BiTes) which, similar to CAR T-cell therapy, activate the endogenous immune system against tumor cells. CD19xCD3 BiTes, blinatumomab, and CD20xCD3 BiTes, epcoritamab, have shown efficacy in pre-clinical studies *in vivo* and *in vitro*.^123,124^ The advantage of BiTes therapy is that it encompasses “off the shelf” products readily available, while CAR T-cells are manufactured for each individual patient. This is potentially advantageous for patients with rapidly progressive disease. However, a one-time treatment approach makes CAR T-cell therapy an appealing option.

Overall, CAR T-cell therapy is a promising therapeutic option for patients with double-refractory (BTKi and BCL2 inhibitors) CLL. Yet, much work needs to be done to optimize this therapy. Thus, determining the optimal time for CAR T-cell therapy administration, improving T-cell fitness to enhance CAR T-cell viability and persistence, choosing the optimal CLL surface antigen(s) to direct the CAR construct against, and further understanding the mechanisms of resistance are pivotal concepts that ought to be further explored in efforts to make CAR T-cell therapy a viable tool in the therapeutic armamentarium against CLL. The high cost of CAR T-cell therapy, as is the case of commercially approved CAR T-cell therapies for DLBCL and MM, and the need for specialized care may limit the broader applicability of this treatment, particularly in developing countries.

### Conflicts of interest

R.M., S.A., M.G., Y.L., R.P., and H.Q.: No COI

M.A.K-D: research/grant support to institutions from Novartis and Bristol Myers Squibb

### Authors’ Contribution per CRediT

Conceptualization: Razan Mohty (Equal), Ricardo Parrondo (Equal), Mohamed A. Kharfan-Dabaja (Equal). Writing – original draft: Razan Mohty (Equal), Shaykha Alotaibi (Equal), Martha Gadd (Equal), Yan Luo (Equal), Ricardo Parrondo (Equal), Hong Qin (Equal), Mohamed A. Kharfan-Dabaja (Equal). Writing – review & editing: Razan Mohty (Equal), Shaykha Alotaibi (Equal), Martha Gadd (Equal), Yan Luo (Equal), Ricardo Parrondo (Equal), Hong Qin (Equal), Mohamed A. Kharfan-Dabaja (Equal). Supervision: Razan Mohty (Equal), Ricardo Parrondo (Equal), Mohamed A. Kharfan-Dabaja (Equal).
